# Pulmonary complications in dogs with acute presentation of pancreatitis

**DOI:** 10.1186/s12917-020-02427-y

**Published:** 2020-06-22

**Authors:** Eleonora Gori, Alessio Pierini, Gianila Ceccherini, Simonetta Citi, Tommaso Mannucci, Ilaria Lippi, Veronica Marchetti

**Affiliations:** Department of Veterinary Sciences, Veterinary Teaching Hospital “Mario Modenato”, University of Pisa, Via Livornese Lato monte, San Piero a Grado, 56122 Pisa, Italy

**Keywords:** Pancreas, Respiratory complications, Arterial, Diagnostic imaging, Canine

## Abstract

**Background:**

In humans, respiratory complications in patients with acute pancreatitis (AP) are a common life-threatening comorbidity. Since possible lung impairment has not been individually evaluated in canine AP, the aims of the present study were to: (1) describe the prevalence, types and severity of pulmonary complications in dogs with acute presentation of AP, and (2) evaluate their association with mortality. AP diagnosis was based on compatible clinical and laboratory parameters, abnormal canine pancreatic-lipase test, and positive abdominal ultrasound within 48 h from admission. The canine acute pancreatitis severity score (CAPS) was calculated for each dog at admission. Arterial blood gas analysis and thoracic radiography were performed at admission. Thoracic radiography was classified on the basis of pulmonary pattern (normal, interstitial or alveolar) and a modified lung injury score (mLIS) was applied to the ventrodorsal projections for each dog. VetALI/VetARDS were diagnosed using current veterinary consensus. Dogs were divided into non-survivors or survivors (hospital discharge). Clinical, radiological and blood gas parameters collected at presentation were compared between survivors and non-survivors and associated with mortality.

**Results:**

This prospective cohort study included twenty-six client-owned dogs with AP. Twelve out of twenty-six dogs (46%) died or were euthanized. At admission, thirteen dogs showed respiratory distress at physical examination, which was associated with death (*P* < 0.001). Radiographic abnormalities were found in twenty-one dogs: alveolar (*n* = 11) and interstitial pattern (*n* = 10). Radiographic alterations and mLIS score were both associated with death (*P* = 0.02 and *P* = 0.0023). The results of the arterial blood-gas evaluation showed that non-survivors had lower PaCO_2_ and HCO_3_^−^ levels, and higher A-a gradient than survivors (*P* = 0.0014, *P* = 0.019 and *P* = 0.004, respectively). Specifically, three dogs had aspiration pneumonia, and VetALI was diagnosed in nine dogs (34.6%), and no dogs met the criteria for VetARDS. The presence of VetALI was associated with mortality (*P* < 0.001).

**Conclusions:**

As with humans, possible lung impairments, such as VetALI, should be investigated in dogs with acute presentation of pancreatitis.

## Background

In humans, pulmonary complications associated with acute pancreatitis (AP) can occur in up to 75% of cases [1, 2]. In veterinary medicine, only a few studies have taken lung impairments into account during canine AP. Hess et al. [3] showed that AP dogs could develop various thoracic abnormalities such as pneumonia (16%), pleural effusion (16%), and pulmonary oedema (6%). Another study included respiratory complications in a clinical severity score for canine AP: dogs with respiratory abnormalities such as dyspnoea, pneumonia or acute respiratory distress syndrome (ARDS) had a higher mortality rate [4]. A more recent study on the occurrence, clinical features and outcome of canine AP [5], failed to find an association between pulmonary impairment and AP in dogs. However, a characterization of the type of respiratory impairment, using clinical signs, radiographic features together with arterial blood gas, was not performed in any of these previous studies.

To date, in humans as in dogs, the diagnosis of acute lung injury (ALI) and ARDS is currently based on the combination of clinical, radiographic, and arterial blood gas abnormalities [6]. Pulmonary impairment may lead to various degrees of respiratory compromise and failure described as ALI and ARDS. Both ALI and ARDS can be caused by the same factors, which are divided into direct and indirect pulmonary damage (e.g., inflammation, infection, systemic inflammatory response syndrome [SIRS], etc.) [6]. The current veterinary consensus [6] established five criteria for the diagnosis of these conditions based on an acute onset of dyspnoea in patients with known risk factors, bilateral diffuse pulmonary infiltrates without evidence of cardiogenic pulmonary oedema, together with evidence of insufficient gas exchange (PaO_2_/FiO_2_ ≤ 300 = VetALI; PaO_2_/FiO_2_ ≤ 200 = VetARDS) [6]. The fifth, and optional criteria, is the evidence of diffuse pulmonary inflammation (transtracheal wash or bronchoalveolar lavage) [6]. Based on the criteria reported above, it is clear that ARDS represents a more severe form of respiratory impairment with a poorer gas exchange than ALI, and thus a more severe form of pulmonary complication which requires more aggressive intervention [6–8].

In humans, ALI and ARDS during AP have a mortality rate ranging from 35% to over 40% [7, 8]. During AP, actively circulating, digestive enzymes cause the release of pro-inflammatory cytokines, activation and migration of leukocytes/neutrophils, and platelet activating factors [9]. In both humans and rats, it has also been hypothesized that AP-associated ALI is also related to phospholipase A2, which correlates with the severity of pulmonary injury [10–12]. Pulmonary parenchyma is damaged as a result of a marked systemic inflammatory response, which is microscopically characterized by increased endothelial and epithelial barrier permeability. This then leads to an exudative phase (days 1–3) with a diffuse alveolar injury, type I pneumocyte necrosis, which causes leakage of a protein-rich exudate into the alveolar and interstitial spaces. Finally, there is a proliferative phase (days 3–7) with lung repair, type II pneumocyte hyperplasia and fibroblast proliferation [9, 12, 13]. In severe AP, ALI and ARDS are one of the main comorbidity factors for early death, especially within the first week after admission, together with single or multiple organ dysfunction [13, 14]. To date, there have been no clinical veterinary studies on the evaluation of pulmonary complications, combining clinical, radiographic and arterial blood gas results in dogs with AP. We hypothesized that, as in humans, during canine AP pulmonary complications, especially ALI and ARDS, may occur and profoundly affect the patient’s prognosis. The aims of the present study were to: (1) describe types and severity of pulmonary complications in dogs with acute presentation of AP, evaluating clinical signs, thoracic x-rays and arterial blood gas parameters, and (2) evaluate their association with mortality and with the severity of the disease.

## Results

Twenty-six client-owned dogs that were admitted to the Veterinary Teaching Hospital from April 2017 to June 2018 were enrolled. The cohort of dogs had a median age of 11.7 years (range 6–16.5 years) which did not differ between survivors and non-survivors (*p* = 0.8). The median body weight was 18.5 kg (range 5–36.5 kg). There were thirteen females (10 spayed), and 13 males (4 neutered) and sex was not associated with mortality (*P* = 0.9). Various dog breeds were represented, with English Springer Spaniel, English Setter and Beagle (*n* = 2 each breed) being the most common, followed by one of each of the following: Standard Poodle, Dachshund, Boxer, Bull Terrier, French Bulldog, English Bulldog, Pug, German Wirehaired Pointer, Golden Retriever, Jack Russell Terrier, Labrador Retriever, Lagotto Romagnolo, German Shepherd, and Bloodhound. Six dogs were mixed breed.

Previously diagnosed comorbidities at the time of inclusion were: hyperadrenocorticism (*n* = 1), diabetes mellitus (*n* = 3), multicentric lymphoma *(n* = 1), chronic GI diseases (*n* = 5) and hepatobiliary diseases (*n* = 3). Each comorbidity was stable and under treatment at the time of the AP diagnosis and no significant difference in mortality between dogs with or without comorbidities was found (*p* = 0.9). Twelve dogs (46%) died or were euthanized (*n* = 2) due to a deterioration in the clinical condition due to progression of the disease. None of the dogs were euthanized due to financial concerns. The overall median CAPS score at presentation was 8 (range 0–15). The median hospitalization time was 2 days (range 1–11 days). Four out of 12 dogs died on day 1 of hospitalization, 3 dogs on day 2. In addition, 2 dogs died at days 3, 2 and 1 dogs died at days 4 and 5 of hospitalization, respectively. None of the dogs underwent mechanical ventilation due to financial concerns, however none of these dogs were euthanized. Three dogs were successfully treated with non-invasive positive airways pressure ventilation using a pediatric helmet with oxygen supplementation. No dogs died during the follow-up period (1 month). Three dogs (11%) with a negative abdominal ultrasound for AP at hospital admission became positive within 2 days of admission.

A comparison of the clinical and radiological signs of pulmonary complication between survivors and non-survivors is reported in Table 1. Briefly, twenty-one dogs out of 26 (80.7%) showed clinical and radiographic signs of pulmonary disease. The respiratory rate was higher in non-survivors (55.6 ± 4.9) compared to survivors (32.0 ± 3.5) (*P* = 0.006). At physical examination, 12 out of 26 dogs (46%) showed respiratory distress and one dog out of 26 (3%) showed cyanosis. Respiratory distress was associated with higher mortality (*P* < 0.001). Twenty-one dogs (80.7%) showed radiographic alterations, which were classified as an alveolar pattern (*n* = 11; 3 lobar and 8 diffuse alveolar pattern) and diffuse interstitial pattern (*n* = 10). Five dogs had no radiographic alterations. The presence of radiographic abnormalities was associated with death (*P* = 0.02).

**Table 1:** Clinical and radiological signs of pulmonary complication in our cohort of dogs

Clinical or radiological signs	Survivors (*n* = 14)	Non-survivors (*n* = 12)	*P*-value
Comorbidities	*n* = 8 (57%)	*n* = 5 (42%)	0.9
Respiratory rate (breaths/minute)	32 ± 3.5	55.6 ± 4.9	**0.006**
Respiratory distress	*n* = 1 (7%)	*n* = 11 (92%)	**< 0.0001**
Radiographic pattern	*n* = 9 (64%)	*n* = 12 (100%)	0.02
Alveolar	*n* = 3 (21%)	*n* = 8 (67%)	
Interstitial	*n* = 6 (43%)	*n* = 4 (33%)	
mLIS			**0.0023**
0	*n* = 5 (36%)	*n* = 0	
1	*n* = 5 (36%)	*n* = 1 (8%)	
2	*n* = 2 (14%)	*n* = 1 (8%)	
3	*n* = 1 (7%)	*n* = 3 (25%)	
4	*n* = 1 (7%)	*n* = 7 (58%)	

*mLIS* Modified lung injury score

**Fig. 1:**
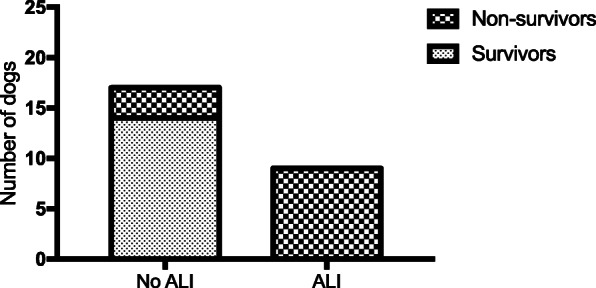
Representative images of four subjects with mLIS score 1 (A), 2 (B), 3 (C) and 4 (D). This figure has to be the figure with 4 x-rays, that now is the Fig. 2

At hospital admission, six dogs had mLIS = 0, five dogs had mLIS = 1, and three and four dogs had mLIS = 2 and 3, respectively. Lastly, eight dogs had mLIS = 4 (Fig. 1). Dogs with higher mLIS score had higher mortality rate (*P* = 0.0023). Dogs with the evidence of pulmonary complications, based on the simultaneous presence of respiratory distress and radiographic abnormalities, had a higher mortality rate than dogs without (*P* = 0.0002). The CAPS score did not differ either between dogs with or without pulmonary complication or between survivors and non-survivors (*P* = 0.053 and *P* = 0.058, respectively).

Arterial blood-gas values for survivors and non-survivors are summarized in Table 2. Three out of 26 (11.5%) showed severe hypoxemia, whereas four dogs showed mild hypoxemia. Non-survivors showed lower PaCO_2_ and HCO_3_^−^ levels than survivors (*P* = 0.01 and *P* = 0.02, respectively). The ROC curve for PaCO_2_ showed that a PaCO_2_ below 25.25 mmHg had a sensitivity of 75% and a specificity of 92.86% in predicting mortality (*P* = 0.01; area under the curve (AUC) = 0.78). In addition, the ROC curve for HCO_3_^−^ showed that a value below 5.3 mmol/L was the optimal cut-off for mortality (*P* = 0.02; sensitivity 83.3%; specificity 85.71%; AUC = 0.77). The PaCO_2_ showed no correlation with the respiratory rate (*P* = 0.54). The alveolar-arterial (A − a) gradient was higher in non-survivors compared to survivor dogs (*P* = 0.04), although the ROC curve was not able to predict the outcome (*P* = 0.08). No differences between groups were found for pH, PaO_2_ and PaO_2_/FiO_2_.

**Table 2:** Arterial blood-gas values in survivors and non-survivors

Parameter	Survivors (*n* = 14)	Non-survivors (*n* = 12)	Reference interval	*P*-value
pH^a^	7.4 (7.14–7.54)	7.4 (7.10–7.76)	7.35–7.46	0.73
PaCO2^a^ (mmHg)	30.75 (13.5–45.9)	23.1 (18–45.5)	34–43.5	**0.014**
PaO2^a^ (mmHg)	97.05 (51.5–120)	89.6 (48.8–110)	80.9–103	.17
A − a gradient^b^ (mmHg)	30.06 ± 11.38	43.86 ± 20.68	0–20	**0.04**
PaO_2_/FiO_2_^a^ (mmHg)	462.1 (245.2–571.4)	426.7 (232.4–523.8)	> 400	.17
HCO_3_^−a^ (mmol/L)	18.45 (5.9–27.1)	13.7 (9.5–36.7)	18–24	**0.019**

^a^Results obtained using Mann-Whitney U-test (data are expressed as median and range in brackets)

^b^Result obtained using unpaired t-test (data are expressed as mean ± standard deviation)

**Fig. 2:**
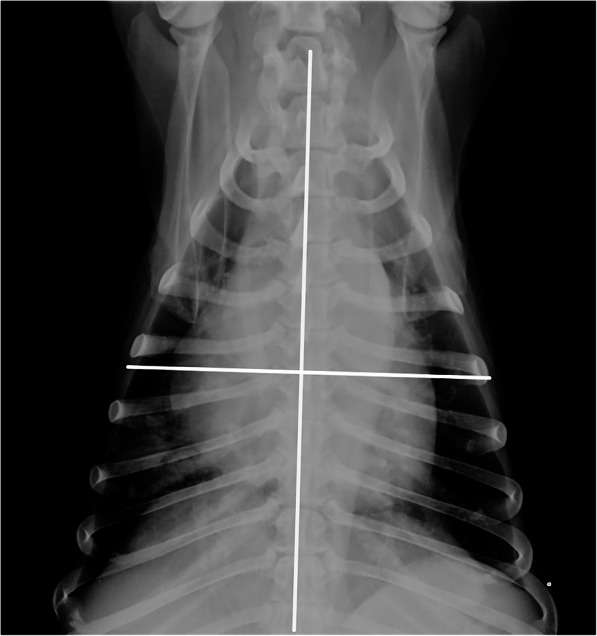
Association between the presence of Acute Lung Injury (ALI) and the outcome (*P* < 0.0001). This figure has to be Fig. 2 but now is registered as Fig. 3

Three dogs (11.5%) had aspiration pneumonia and no dogs showed pleural effusion. ALI was diagnosed in nine dogs (34.6%) and no dogs showed ARDS. The presence of ALI was associated with higher mortality (*P* < 0.001; Fig. 2).

## Discussion

To the best of our knowledge, this is the first report to describe the types and severity of pulmonary complications in dogs with acute presentation of AP, evaluating the clinical signs, thoracic x-rays and arterial blood gas parameters, and also evaluating their association with mortality and the severity of the disease.

As reported in humans with AP, our findings showed a relatively high prevalence of pulmonary complications in the dogs enrolled. In fact, half of the study population showed respiratory distress, and over 80% of dogs showed radiographic abnormalities. In particular, the presence of radiographic abnormalities was associated with a higher mortality.

In human medicine, the clinical presentation of respiratory complications in AP include: (1) pulmonary manifestations without any radiological abnormality, which may show hypoxemia, tachypnoea and mild respiratory alkalosis; (2) radiological changes, such as pulmonary infiltrates or atelectasis (15%), pleural effusions (4–17%), and pulmonary oedema/ARDS (8–50%) along with hypoxemia; and (3) clinically relevant ALI/ARDS [9]. Based on the results of our study, it seems that many dogs may belong to the second group, in which there are radiographic abnormalities, but also some arterial blood gas alteration (e.g., increased A-a). However, in canine AP also radiographic abnormalities, and in most severe ALI cases, may occur (e.g., alveolar pattern). To date, in the veterinary literature, respiratory alterations have rarely been studied and have not been investigated individually. One study investigating a clinical severity index for canine AP showed that respiratory alterations were associated with mortality [4]. In this latter study, 4 out of 61 dogs (6.6%) showed clinical evidence of dyspnoea or tachypnoea, and respiratory alterations were associated with higher mortality rates. In another study [5], 23% of dogs (*n* = 18) showed tachypnoea and/or dyspnoea, which was not associated with the outcome. Another study investigating the histological association between lung inflammation and AP in dogs, concluded that, despite clinical evidence suggesting pulmonary complications in only 4 out of 21(19%) dogs, most dogs with AP showed histological pulmonary inflammation and increased levels of inflammatory markers [15]. Lastly, a very recent study on the prognostic factors of canine AP concluded that respiratory complications can negatively influence the patient’s prognosis, however, based on their results, respiratory distress was not associated with mortality [16]. During AP, tachypnoea can be associated with abdominal pain and metabolic acidosis, fever, opioid administration, and at the same time it has been shown to be caused by pulmonary parenchymal disease, such as aspiration pneumonia or VetALI/VetARDS [17]. However, none of our dogs had hyperthermia or received opioids prior to presentation.

In the present study, a modified LIS was used to evaluate the extension of the pulmonary disease, based on how many thoracic areas were involved. Non-survivors showed a higher mLIS and a higher frequency of pulmonary radiographic pattern compared to survivors. In 1988, Murray and colleagues developed an LIS, which took into account thoracic radiographs in addition to a hypoxemia score, positive end-expiratory pressure and static compliance of the respiratory system. Our results agree with Murray’s and other studies which showed that the more extensive the pulmonary disease, the higher the risk of death [9, 17–20].

Non-survivors showed lower PaCO_2_ and HCO_3_^−^ levels than survivors. In humans, arterial blood gas parameters are not evaluated to predict the severity and outcome in AP, although some of the parameters (pH, PaO_2_) are included in some scoring systems, such as Ranson’s and the Acute Physiology and Chronic Health Examination [21, 22]. Sharma et al. [23] showed how patients with metabolic acidosis and low HCO_3_^−^ levels were more likely to develop organ failure and had higher mortality rates. Up to 65% of the patients who developed respiratory failure had bicarbonate levels lower than the reference range [29]. To the best of our knowledge, there is only one study [24] that has investigated arterial blood gas in dogs with experimentally induced AP. Uchikov and colleagues evaluated only changes in the arterial oxygen levels and acid-base status at various stages of the disease. However, they found that all dogs with AP showed persistently low levels of PaCO_2_ at different time points [24]. A decrease in PaCO_2_ levels could be due to primary or secondary respiratory diseases resulting in an increase in hyperventilation [17]. One reason for this finding could be the higher respiratory rate in non-survivors caused by more severe abdominal pain compared to survivors due to more severe forms of AP [3, 25]. Alternatively, as supported by our results, hyperventilation may be secondary to an attempt to compensate for a metabolic acidosis, which is a fairly common condition in both human and canine AP [17, 26]. Moreover, hypocapnia and low pH stimulate central and peripheral chemoreceptors, which act directly on the brainstem with an increased in respiratory rate [26].

Our results showed a higher A-a gradient in non-survivors than in survivors despite a median normal value of PaO_2_ in both groups (Tab.1). The A-a gradient is a clinically useful method to evaluate the degree of pulmonary parenchymal disease [27] and, based on our results, it was shown to have a good sensitivity in detecting pulmonary interstitial/parenchymal abnormalities. As PaCO_2_ is taken into consideration in the equation for the calculation of the gradient, hypoventilation was ruled out as a potential cause of hypoxemia in our patients (only one dog had PaCO_2_ > 45 mmHg). High values of the A-a gradient can be found in various forms of pulmonary parenchymal disease (from ventilation-perfusion mismatch and shunting) or resistance to the diffusion of oxygen across the alveolar membrane [27]. Our findings may be due to the higher prevalence of clinical and/or radiological lung diseases in the non-survivors, resulting in a higher A-a gradient in this group.

Based on current veterinary consensus [6], ALI was diagnosed in nine dogs (34.6%) and no dogs developed ARDS. The presence of ALI was associated with poor outcome. ALI and ARDS are considered to be the primary cause of death in the early stage of AP in humans. Up to 15–20% of AP patients develop ARDS with an associated mortality of 35–50% [7]. In dogs, AP is reported to be one of the risk factors for the development of VetALI/VetARDS [20, 28]. In 1995, one case report described a dog with histologically confirmed AP, which developed marked expiratory dyspnoea, and ARDS was diagnosed on the detection of radiographic bilateral pulmonary infiltrates and histologic evidence of pulmonary inflammation [29]. However, there has been no detailed evaluation of VetALI/VetARDS during AP in the literature. To date, in dogs there are no studies on the pathophysiology of lung injury during AP, and the current literature is based on studies performed on rats and on humans, in which pancreatic enzymes were related to direct pulmonary injury [10–12, 30].

The present study has some limitations. One limitation is the low number of dogs enrolled. Due to our stringent inclusion criteria, only a small population of dogs could be included. Secondly, as a preliminary study, we did not recheck the arterial blood-gas or thoracic radiography, which could be the focus of a larger-scale prospective study. Finally, no dogs had undergone a necropsy examination, which is essential for a histological characterization of both pancreatic and pulmonary diseases.

## Conclusions

As in humans, AP is a potentially life-threatening disease, which can lead to several systemic complications, including pulmonary diseases. In our dogs with AP, approximately half showed respiratory distress, and over 80% showed radiographic alterations. This was especially the case for non-survivors which had a higher mLIS compared to survivors, thus showing how pulmonary complications may be frequent and should be monitored by clinicians. Lower PaCO2 values and a higher A-a gradient were associated with a higher mortality rate in dogs with AP. This thus shows that arterial blood gas analysis may be a useful prognostic tool in these patients, together with a clinical and diagnostic imaging evaluation. VetALI was diagnosed in nine dogs and was associated with death. Further large-scale studies are warranted to investigate early markers that are capable of predicting the outcome and facilitating a prompt treatment.

## Methods

### Case selection criteria

Dogs in this study were enrolled with the owners’ informed consent and with the approval of the Ethics and Welfare Committee (Approval No. 16749/2017).

Over a one-year period, from April 2017 to June 2018, client-owned dogs with AP that had been referred to our veterinary teaching hospital, were prospectively enrolled.

The following data were recorded for all dogs: signalment, physical examination findings and comorbidities. The diagnosis of AP was based on: (1) two or more of the following clinical signs: abdominal pain, diarrhoea, vomiting or anorexia/inappetence; (2) abdominal ultrasonographic (Xario GC, Toshiba, Japan) findings consistent with AP within 48 h from hospital admission without other identifiable extra-pancreatic diseases and (3) an abnormal result with rapid point-of-care semiquantitative canine pancreatic lipase immunoassay (Idexx SNAP® cPL™, Idexx Laboratories, Milan, Italy).

Patients were excluded if cardiogenic causes of respiratory distress could not be definitively ruled out. Dogs with a clinical history of chronic respiratory signs were also excluded.

All abdominal ultrasound scans were performed by the same radiologist (TM) and were consistent with AP diagnosis if the following were present: hypoechoic and enlarged pancreatic parenchyma, hyperechoic mesenteric areas around the pancreas and peripancreatic effusion [25]. Dogs with clinical and clinicopathological features consistent with AP, but without a positive abdominal ultrasound, were rechecked every 24 h and included if they developed ultrasonographic findings compatible with AP within 2 days of their admission. For each dog, the previously validated canine acute pancreatitis severity score (CAPS) was calculated at admission [31].

### Evaluation of pulmonary complications

Pulmonary complications were defined if evidence of both respiratory distress, defined as the presence of tachypnoea (> 40 breaths/min) and laboured breathing at rest, and thoracic radiographic abnormalities were present (presence of a lung pattern) [1].

Arterial blood gas analyses were performed for each dog at admission, without oxygen supplementation or administration of sedatives. Dogs presented with clinical evidence of respiratory distress at the triage evaluation were admitted to the intensive care unit for an emergency evaluation. In dogs that did not tolerate restraint and handling, or animals with known coagulopathy, arterial blood gas was not performed at presentation at room air, but flow-by oxygen supplementation was administered first. These subjects were consequently excluded from the study.

The arterial blood gas analysis was performed from the dorsal pedal artery in order to assess the initial oxygenation. Arterial blood samples (approximately 0.4 mL) were obtained for each patient at room air (FiO_2_ 0.21%) using a 60 IU balanced heparin self-filling sampler (*safe*PICO arterial blood gas syringe, Radiometer Medical, Copenhagen, Denmark), which was run immediately in cooximetry mode using a blood gas analyser (ABL 700 series, Radiometer Medical, Copenhagen, Denmark).

Digital thoracic radiographs were performed for each dog after the initial clinical respiratory stabilization in both right and left lateral and ventrodorsal recumbency using digital radiological equipment (Multimage HF Cosmovet, Cavaria, Italy). Subsequently all the DICOM files were blindly reviewed by a radiologist (SM) and evaluated based on the pulmonary pattern (normal, interstitial, or alveolar). The presence of pleural effusion and other significant findings were also recorded. An alveolar lung pattern was defined as the presence of one or more of the following radiographic features: (1) air bronchogram, (2) lobar sign, or (3) area of increased soft tissue opacity without sharp margins [32]. The diagnosis of an unstructured interstitial pattern was based on the finding of an abnormal increase in the background radiographic opacity of the lung with blurred vascular margins.^11^ Lastly, pleural effusion was diagnosed if there were: widened interlobar fissures with soft tissue opacity, retraction of pleural surface of lung that was not directly on the pleural surface of thoracic wall, increased soft tissue opacity with scalloped margins dorsal to sternum (in lateral projections), decreased cardiac silhouette visualization, and obscured diaphragmatic outline [32].

**Fig. 3:**
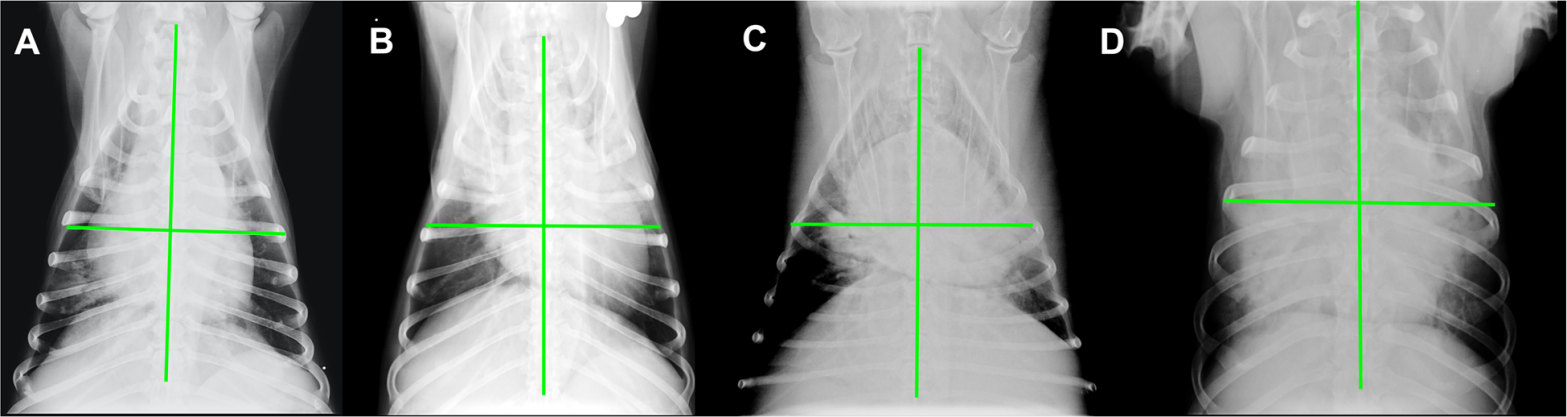
Subdivision of the thorax into four lung quadrants as defined in the modified lung injury score (mLIS). The mLIS was calculated based on how many quadrants were radiographically involved. This figure has to be the one with the single x-ray, which now is Fig. 1

The modified Murray lung injury score [18] (mLIS) for radiographic pulmonary alterations was applied using ventrodorsal projection, as previously used in veterinary medicine [19, 20]. The mLIS ranged from 0 to 4 points as previously described [19, 20]. The VD projection was divided into four overall quadrants: right cranial, right caudal, left cranial, and left caudal using a vertical line starting in the middle of the trachea from the thoracic inlet and continuing through the middle of the thorax, and a horizontal line perpendicular to the vertical line through the centre of the sternum (Fig. 3).

VetALI or VetARDS were diagnosed using the current veterinary consensus (Table 3) and, based on the PaO2/FiO2 ratio (VetALI ≤300 mmHg and VetARDS ≤200 mmHg, respectively) [6]. Aspiration pneumonia was diagnosed if a dog had: (1) a condition predisposing to aspiration (e.g. vomiting and regurgitation), (2) compatible respiratory signs (e.g. tachypnoea, dyspnoea, increased respiratory effort, crackles or wheezes at lung auscultation and/or coughing), and (3) compatible x-ray pattern (patchy or focal alveolar pattern above all in the ventral portion of the cranial lobes, more rarely in the middle lung lobe) [33]. The final outcome for each dog was classified as death (non-survivors) or discharge from the hospital (survivors). Euthanasia was performed after the owner’s informed consent and if there was a deterioration in the clinical condition due to a progression of the respiratory disease. However, invasive or non-invasive positive pressure ventilation through a pediatric helmet (Dimar Medical Devices Srl, Medolla, MO, Italy. www.dimarsrl.com) was always proposed if necessary [34].

**Table 3:** Definition of VetALI/VetARDS: Veterinary Acute Lung Injury and Acute Respiratory Distress Syndrome [25]

Must meet at least one each of the first four criteria; 5 is a recommended but optional measure	
**1**. Acute onset (72 h) of tachypnoea and laboured breathing at rest. **2**. Known risk factors (inflammation, infection, sepsis, SIRS, severe trauma, multiple transfusions, smoke inhalation, near-drowning, aspiration of stomach contents, drugs and toxins). **3**. Evidence of pulmonary capillary leak without increased pulmonary capillary pressure^a^ (any one or more of the following): a. Bilateral/diffuse infiltrates on thoracic radiographs (more than 1 quadrant/lobe) b. Bilateral dependent density gradient on CT c. Proteinaceous fluid within the conducting airways d. Increased extravascular lung water **4**. Evidence of inefficient gas exchange (any one or more of the following): a. f. Hypoxemia without PEEP or CPAP and known FiO2 i. PaO_2_/FiO_2_ ratio (**≤**300 mmHg for VetALI; **≤**200 mmHg for VetARDS) ii. Increased alveolar-arterial oxygen gradient iii. Venous admixture (non-cardiac shunt) b. Increased ‘dead-space’ ventilation **5**. Evidence of diffuse pulmonary inflammation a. Transtracheal wash/bronchoalveolar lavage sample neutrophilia b. Transtracheal wash/bronchoalveolar lavage biomarkers of inflammation c. Molecular imaging (PET)	

^a^No evidence of cardiogenic oedema (one or more of the following):

No clinical or diagnostic evidence supporting left heart failure, including echocardiography

*CT* Computed tomography; *PEEP* Positive end expiratory pressure; *CPAP* Continuous positive airway pressure; *FiO2* Fraction inspired oxygen; *PET* Positron emission tomography

Since there are no consensus statements on VetALI/VetARDS treatment [35], at the time of the diagnosis, prompt treatment with non-invasive continuous positive airway pressure and low dose dexamethasone (0.1 mg/kg SID) and antibiotics (ampicillin at 22.5 mg/kg TID and marbofloxacin at 2 mg/kg SID) when needed (aspiration pneumonia) were performed.

### Statistical analysis

For all continuous parameters, the normality of data distribution was evaluated by the D’Agostino-Pearson test. Normally and non-normally distributed continuous parameters were reported as mean ± standard deviation (SD), and as median and range, respectively, and were compared between survivors and non-survivors using the t-test or Mann-Whitney test, respectively. Fisher’s exact test, or Chi-square test, were used to compare categorical variables. As stated above, mortality was evaluated at hospital discharge for each dog and dogs were divided into two groups: survivors (discharged from the hospital) and non-survivors. For each dog a telephone or clinical follow-up was performed at least 1 month after the discharge. Respiratory distress, respiratory rate, the presence of radiographic abnormalities, and the presence of pulmonary complications (aspiration pneumonia, pleural effusion, VetALI/VetARDS) were compared to outcomes. Age, pH, PaCO_2_, PaO_2_, Alveolar-arterial (A − a) gradient, PaO_2_/FiO_2_, HCO_3_^−^ and mLIS were evaluated between survivors and non-survivors using the Mann-Whitney U-test or unpaired t-test based on the normality distribution. The CAPS score was compared between dogs with or without pulmonary complications using the Mann-Whitney U-test. A receiver operating characteristic (ROC) curve was plotted for any parameter that was significantly different between survivors and non-survivors in order to find the optimal cut-off for mortality. A Pearson’s correlation test was performed between the respiratory rate and PaCO_2_. Mild hypoxemia was defined as PaO_2_ < 80 mmHg, and severe hypoxemia as a PaO_2_ < 60 mmHg.^31^ Data were analysed with Graphpad Prism 7 (GraphPad Software Inc., La Jolla, CA, USA). A *P*-value of < 0.05 was considered significant.

## Data Availability

The datasets used and/or analysed during the current study are available from the corresponding author on reasonable request.

## Abbreviations

AP: Acute pancreatitis
ALI: Acute lung injury
ARDS: Acute respiratory distress syndrome
AUC: Area under the curve
mLIS: Modified lung injury score
SD: Standard deviation
A-a: Alveolar-arterial
